# Comparative Study on Phenolic Content and Antioxidant Activity of Different Malt Types

**DOI:** 10.3390/antiox10071124

**Published:** 2021-07-14

**Authors:** Vesela Shopska, Rositsa Denkova-Kostova, Mina Dzhivoderova-Zarcheva, Desislava Teneva, Petko Denev, Georgi Kostov

**Affiliations:** 1 Department of Technology of Wine and Beer, University of Food Technologies—Plovdiv, 26 Maritza boulevard, 4002 Plovdiv, Bulgaria; vesi_nevelinova@abv.bg; 2 Department of Biochemistry and Molecular Biology, University of Food Technologies—Plovdiv, 26 Maritza boulevard, 4002 Plovdiv, Bulgaria; rositsa_denkova@mail.bg; 3 Department of Technology of Tobacco, Sugar, Vegetable and Essential Oils, University of Food Technologies—Plovdiv, 26 Maritza boulevard, 4002 Plovdiv, Bulgaria; minadj@abv.bg; 4 Laboratory of Biologically Active Substances, Institute of Organic Chemistry with Centre of Phytochemistry, Bulgarian Academy of Sciences, 139 Ruski boulevard, 4000 Plovdiv, Bulgaria; desislava.teneva@orgchm.bas.bg (D.T.); petko.denev@orgchm.bas.bg (P.D.)

**Keywords:** malt, antioxidant capacity, principal component analysis, phenolic compounds, 2,2′-diphenyl-1-picrylhydrazyl (DPPH), 2,2′-azinobis-(3-ethylbenzothiazoline-6-sulfonate (ABTS), ferric reducing ability of plasma (FRAP), cupric reducing antioxidant capacity (CUPRAC), Oxygen Radical Absorbance Capacity (ORAC)

## Abstract

Malt is the main raw material for beer production, which determines not only its taste and aroma profile, but to a large extent its biological value, as well. The aim of the present research was to determine the antioxidant profile of different malt types as a basis for the development of new types of beer with increased antioxidant activity. In the present study the main brewing characteristics, the phenolic profile and the antioxidant potential of 20 malt types used in craft breweries in Bulgaria have been examined. The main brewing characteristics have been determined by the standardized methods of the European Brewing Convention. Malt phenolic content was determined by two methods, and antioxidant potential by five different methods. Based on a statistical factor analysis performed by the principal component analysis, it was confirmed that there was a relationship between malt color and phenolic compounds content. The principal component analysis confirmed that there was a link between the content of the Maillard reaction products and malt biological activity. Malts with the highest degree of heat treatment were characterized by the highest antioxidant activity, which was due to the content of Maillard reaction products with antioxidant capacity.

## 1. Introduction

Beer production involves the use of four main raw materials—malt, hops, water and brewer’s yeast. The main sources of starch in brewing are different types of barley malt, wheat malt, corn, unmalted barley, sorghum and others [1,2]. Malt not only provides the necessary amount of starch for the production of fermentable sugars and the color of the final beer, but also contributes to the oxidative stability of the beverage through its content of phenolic compounds and antioxidants [3,4]. Various studies have shown that malt provides about 80% of the total amount of phenolic compounds in beer, and the remaining 20% are due to the hops used [3,5,6]. At the same time, malt through its components, provides between 86% and 95% of the total antioxidant potential of beer [3,7]. It has been found that hops do not significantly affect the phenolic content and antioxidant potential of beer [3,8].

The main changes in the phenolic profile and antioxidant potential of malt occur during its germination, kilning and eventual roasting. Kilning and roasting lead to changes in the phenolic content and the enzyme activity and cause non-enzymatic browning with a significant effect on the overall malt antioxidant properties [3,7].

Barley and malt are rich in phenolic compounds—flavan-3-ols, proanthocyanidins, hydroxycinnamic acid derivatives and small amounts of flavanols. These compounds are found in both bound and free form. Flavan-3-ols are most commonly identified from the free phenolic compounds, and the bound forms are most commonly phenolic acids. A number of authors noted that the most common representatives of this group are catechin and ferulic acid [3,8,9,10,11,12,13,14]. A number of authors point out the fact that the malt production process leads to a decrease in the amounts of catechin, prodelfinidine B3, procyanidin B3 and ferulic acid in the grain [3,8,11,15,16]. Ferulic acid has been found to be more stable and as a result is found in larger amounts in the final malt [3,8]. The malting process and mainly the kilning step of green malt led to an increase in the amount of esterified fractions of phenolic compounds [3,12]. These changes are associated with enzymatic release of bound phenolic compounds, as well as glycosylation reactions. This in turn leads to their easier extraction in the process of obtaining wort [3,12,16,17,18].

The malt heat treatment during kilning and roasting leads to different types of changes. In some cases, degradation of phenolic compounds is observed, in other cases their polymerization. In most cases, kilning leads to a decrease in the ferulic acid amount, which is associated with the inclusion of phenolic compounds in the structure of the formed melanoidins. The process of reducing the amount of ferulic acid is also associated with the thermal degradation of the esterase responsible for the release of ferulic acid [3,18,19].

Thermal treatment of malt during kilning is associated with the formation of products of the Maillard reaction [3,20,21,22,23]. Depending on the ratio of reducing sugars and amino acids in green malt, different types of complex compounds are obtained. In addition, the content of reaction products is strongly influenced by the temperature and the duration of the heat treatment. It has been found that the malt roasting processes lead to active pyrolytic and degradation processes and increased accumulation of high molecular weight components with brown color [3,22,23,24,25]. These differences in heat treatment affect the quality of the malts. Pale and caramel malts are characterized by the content of low molecular weight colorants, while roasted malts are characterized by content of high molecular weight colorants, known as melanoidins [3]. They have high reduction potential and an intense brown color, which is responsible for the color developed of the roasted malts [3,24].

The malt antioxidant potential plays an essential role in beer stability during storage. It has been found that beer sensory properties change during storage as a result of various chemical reactions [3,4,26]. Malt antioxidants play an important role in maintaining the beer oxidative stability, but are also important for consumer health, namely by preventing and neutralizing ROS associated with many diseases: cancer, cardiovascular and other diseases [27]. The antioxidant potential of malt and beer is most often associated with phenolic compounds. Phenolic acids are distinguished due to their ability to donate hydrogen and electrons, as well as to form stable radical intermediates with the greatest antioxidant potential. However, compounds with a flavonoid structure generally show higher antioxidant activity than non-flavonoid compounds [3,19,28,29,30]. In addition to the phenolic compounds, a large part of the Maillard reaction products also show strong antioxidant potential. The data show that the total antioxidant potential of malt increases during kilning and roasting, which is associated not only with the release of phenolic compounds, but also with the formation of reaction products and reductones [3,4,16,18,20,22].

One of the main difficulties comes from the question: How to distinguish malts in terms of their phenolic capacity and antioxidant potential. As it has already become clear, these two parameters are related and also depend on a number of factors—barley variety, malt production technology, storage conditions and others. In this sense, a complex database, which should be interpreted in an appropriate way, is obtained. Principal Component Analysis (PCA) is a factor analysis method that achieves better interpretation of the data and reduces the number of dimensions in the factor space. This is achieved by creating new variables known as principal components and looking for a relationship between the observed values and the principal components. The method is adaptive and allows the classification of non-numerical variables (such as malt type) into separate groups based on numerical variables, i.e., the method can be adapted to different types and structures of data [31].

The aim of the present work was to analyze the phenolic content and antioxidant activity of different types of malt, used in the brewing industry as a basis for the development of new types of beer with increased biological value. By determining the main brewing characteristics of different malt types, their phenolic complex and their antioxidant potential, malts have been grouped into 3 different groups—basic malts, special malts and functional malts. An approach for the development of new types of beer with increased biological potential will be proposed based on the results obtained.

## 2. Materials and Methods

### 2.1. Malt

The following malt types produced by BestMalz, Kurfürsten-Anlage 52, D-69115 Heidelberg, Germany: Acidulated malt; Pilsen malt; Pale ale malt; Munich dark malt; Vienna malt; Smoked malt; Rye malt; Red X malt; Caramel amber malt; Caramel hell malt; Black malt; Melanoidine malt; Wheat malt; Caramel pils malt; Munich malt; Special X malt; Special Wheat malt; Caramel Munich I malt; Caramel Munich II malt; Chocolate malt were used in the present research.

### 2.2. Determination of the Main Characteristics of Malt and Wort

#### 2.2.1. Mashing Method

A standard EBC method was used to determine the malt brewing characteristics. 50 g of the corresponding ground malt were mixed well with 200 mL of water at a temperature of 45–46 °C to obtain a homogeneous mixture. The mixture was placed on a mashing apparatus and mashed for 30 min at 45 °C. After that, the mash temperature was risen to 70 °C with a heating rate of 1 °C/min. Upon reaching 70 °C, 100 mL of water of the same temperature was added to the slurry. The mixture was mashed at 70 °C for 60 min. After that, the samples were cooled for 10–15 min, then diluted to 450 g with water and filtered through Machery-Nagel 614 1/4 filter paper. During filtration, the first 100 mL of the filtrate were returned for re-filtration. Analyzes of the brewing characteristics and the biological value of the malts were performed using the wort obtained [32]. 

#### 2.2.2. Main Characteristics of Malt and Wort

A. Yield of extract—according to method 4.5.1 [32]

B. Malt color—according to the catalog data provided on the manufacturer’s website. 

C. Moisture content of malt—according to method 4.2 [32]

D. Starch content in malt.

The malt was ground and a 3–5 g sample was weighed, transferred quantitatively into a 100 cm^3^ volumetric flask, then 25 cm^3^ of 1% HCl was added. The mixture was stirred by shaking and another 25 cm^3^ of 1% HCl was added. The sample was placed in a boiling water bath for 15 min, stirring continuously for the first 3–5 min. Under these conditions, the starch turns into a soluble form due to its partial hydrolysis. After removal from the water bath, 30 cm^3^ of distilled water was added to the flask and, after cooling, 5 cm^3^ of phosphoric-tungstic acid, which precipitates the proteins, was added. The sample was then diluted to 100 cm^3^ with distilled water, homogenized and filtered through a dry folded filter. The starch content was calculated according to Equation (1) [33].

(1) $$\mathrm{St}=\frac{100\times \alpha \times 100\times 100}{\left[\alpha_{Д}^{20}\right]}\%$$

where: St—starch content, %; α—specific angle of rotation of the starch sample; $\left[\alpha_{Д}^{20}\right]$—specific angle of rotation of pure starch obtained from malt, measured at standard conditions.

### 2.3. Extraction and Determination of Phenolic Compounds

#### 2.3.1. Extraction of Phenolic Compounds from Malt and Wort

10 g of ground malt (of a size corresponding to the requirements of Method 4.5.1 of Analytica EBC) was mixed with 40 cm^3^ of 80% (*v/v*) methanol. After an overnight stay, the mixture was diluted to 50 cm^3^ with the methanol solution. The resulting extract was filtered through a filter (Whattman No.1) and stored at −20 °C until use.

The wort obtained according to method 2.1 was diluted with methanol (100%) in a ratio of 1:10, left to stand for 30 min and filtered using Whattman No.1 filter paper.

The obtained extracts of malt and wort were used to determine the content of phenolic compounds and the antioxidant potential of malt and wort.

#### 2.3.2. Determination of Phenolic Compound Content in Malt and Wort

##### A. Content of Total Phenolic Compounds with FC-Reagent

The content of total phenolic compounds was determined by the Folin-Ciocalteu (FC) method [12] with the following modifications. 1 cm^3^ of methanol extract, 4 cm^3^ of Folin-Ciocalteu working solution, 5 cm^3^ of sodium carbonate (7.5%, *w/v*) were mixed in a test tube. The mixture thus obtained was stirred and allowed to stand for 1 h, after which the absorbance of the sample at 765 mn was determined on a Shimadzu UV-VIS1800 spectrophotometer (Kyoto, Japan) against a blank prepared with distilled water. The results were presented as gallic acid equivalent in mg GAE/dm^3^ wort:

(2) $$\mathrm{TPC}=\frac{\left(A_{765}\;+\;0.0083\right)}{0.0098}\mathrm{Kp},{\;\mathrm{mg}\;\mathrm{Galic}\;\mathrm{acid}/\mathrm{dm}}^{3}$$

##### B. Content of Phenolic Compounds by the Glories Method

The content of total phenolic compounds, phenolic acids and flavonoid phenolic compounds was determined by a modified method of Glories [34]. 1 cm^3^ of 0.1% HCl in 95% ethanol (*v/v*), 18.2 cm^3^ of 2% HCl (*v/v*) and 1 cm^3^ of methanol extract were mixed in a tube. The solution was stirred thoroughly and allowed to stand for 15 min, after which the absorbance was measured against a blank prepared with distilled water. The absorbance (A) at 280 nm was used to evaluate the total phenolic content, A_320_ nm was used to evaluate the content of phenolic acids and A_360_ nm was used to evaluate the content of flavonoid phenolic compounds. The calibration curves for the total phenolic compounds, phenolic acids and flavonoid phenolic compounds were constructed using gallic acid, caffeic acid and quercetin, respectively:

(3) $$\mathrm{TPC}=391.88\times A_{280}\times \mathrm{Kp},\;\mathrm{mg}\;\mathrm{Galic}\;\mathrm{acid}/L$$

(4) $$\mathrm{PA}=210.83\times A_{320}\times \mathrm{Kp},\;\mathrm{mg}\;\mathrm{Caffeic}\;\mathrm{acid}/L$$

(5) $$\mathrm{FPC}=321.94\times A_{360}\times \mathrm{Kp},\;\mathrm{mg}\;\mathrm{Quercetin}/L$$

#### 2.3.3. Antioxidant Potential of Malt and Wort

##### A. Antioxidant Activity Against the DPPH (2,2′-Diphenyl-1-picrylhydrazyl) Radical

The antioxidant activity of malt and wort was measured by the DPPH method [35] with some modifications. 250 μL of the methanol extract was added to 2.25 cm^3^ of DPPH solution in methanol (6 × 10^−5^ M); the mixture was left for 15 min (kept in the dark at room temperature) to allow the reaction to proceed and then the absorbance at 517 nm against a blank of purified water was determined. The control sample was prepared with methanol. The antioxidant activity was determined by a standard curve using Trolox as standard, and the results were expressed as μM Trolox equivalents per 1 dm^3^ for wort:

(6) $$\mathrm{DPPH}\%=100\times \frac{\left(A_{517}^{k}-A_{517}\right)}{A_{517}^{k}},\;\%\;\mathrm{inhibition}$$

(7) $$C_{\mathrm{TROLOX}}=\frac{0.6711+\mathrm{DPPH}\%}{0.341}$$

(8) $$\mathrm{DPPH}=K_{P}\times C_{\mathrm{TROLOX}},{\;\mu \mathrm{mol}\;\mathrm{TROLOX}/\mathrm{dm}}^{3}$$

##### B. Antioxidant Activity by the FRAP (Ferric Reducing Ability of Plasma) Method

The FRAP analysis was performed according to the method of Benzie and Strain (1996) [36] with the following modifications. The stock solution was 300 mM acetate buffer (3.1 g of C_2_H_3_NaO_2_ • 3H_2_O and 16 cm^3^ of C_2_H_4_O_2_) with a pH of 3.6, 10 mM TPTZ solution prepared in 40 mM HCl and 20 mM FeCl_3_ • 6H_2_O solution. The working solution was prepared by mixing the acetate buffer, TPTZ solution and FeCl_3_ • 6H_2_O solution in a ratio of 10:1:1. 150 μL of the methanol extract was allowed to react with 2850 μL of FRAP solution for 4 min in the dark. The absorbance of the sample was then measured at 593 nm against a blank prepared with methanol. The antioxidant activity was determined by a standard curve using Trolox as standard, and the results were expressed as μM Trolox equivalents per 1 dm^3^ for wort:

(9) $$\mathrm{FRAP}=\frac{A_{593}+0.0235}{0.0024}K_{P},{\;\mu \mathrm{mol}\;\mathrm{TROLOX}/\mathrm{dm}}^{3}$$

##### C. Antioxidant Activity by the ABTS (2,2′-Azinobis-(3-ethylbenzothiazoline-6-sulfonate)) Method

The ABTS analysis was performed according to the method of Iqbel et al. (2015) [37] with the following modifications. 7 × 10^−3^ M ABTS solution and 2.45 × 10^−3^ M potassium persulfate solution were prepared. The two solutions were mixed in a ratio of 1:1 and the mixture was left in the dark for 12–16 h. The resulting solution was stable for 2 days. It was then diluted with methanol in a ratio of 1:30 until an absorbance of 1.1 ± 0.1 was reached, which was measured against methanol at λ = 734 nm. The methanolic extracts of malt and wort were diluted with deionized water in an extract: water ratio of 0.5 cm^3^ + 9.5 cm^3^ or 1 cm^3^ + 9 cm^3^, or other suitable dilution to achieve A_734_ in the range of 0.2–0.9. A blank sample (methanol), a control sample—0.15 cm^3^ MeOH + 2.85 cm^3^ ABTS and a working sample—0.15 cm^3^ of diluted extract + 2.85 cm^3^ ABTS were prepared. The measurements were made at λ = 734 nm and the results were calculated based on the dependence:

(10) $$I=\frac{A_{1}-A_{2}}{A_{1}}\times 100,\;\%$$

where: A_1_—the absorbance recorded when measuring the control against the blank sample; A_2_—the absorption recorded when measuring the working sample against the blank sample;

Depending on the percentage of inhibition (I, %), the concentration of Trolox solution (C_Trolox_) by the equation of the standard curve was determined. The antiradical activity, expressed as mM Trolox for L extract (mM TE/dm^3^) was determined after 120 min:

(11) $$C_{\mathrm{Trolox}}=\frac{I+1.6762}{0.1164},\;\mu \mathrm{mol}\;\mathrm{TE}$$

(12) $${\mathrm{AOA}}_{\mathrm{ABTS}}=K_{p}\times C_{\mathrm{Trolox}},{\;\mu \mathrm{mol}\;\mathrm{TE}/\;\mathrm{dm}}^{3}$$

K_p_ = K_MeOH_ × K_water_ (13)

where: K_p_—dilution coefficient of the extract (13).

##### D. Antioxidant Activity by the CUPRAC (Cupric Reducing Antioxidant Capacity) Method

The CUPRAC analysis was performed according to the method of Apak et al. (2006) [38]. The following solutions were prepared—0.01 M solution of CuCl_2_·2H_2_O, acetate buffer with pH = 7 and 7.5 × 10^−3^ M solution of neocuproin. The wort was diluted to a suitable dilution with methanol. If necessary, further dilution of the methanol extracts with deionized water was performed before the analysis.

The following samples are prepared: a working sample—1 cm^3^ of CuCl_2_·2H_2_O + 1 cm^3^ of acetate buffer + 1 cm^3^ of neocuproin + 0.5 cm^3^ of extract + 0.6 cm^3^ of distilled water; a blank sample—1 cm^3^ of CuCl_2_·2H_2_O + 1 cm^3^ of acetate buffer + 1 cm^3^ of neocuproin + 1.1 cm^3^ of distilled water. The samples were homogenized and allowed to stand for 30 min at room temperature. Measurement of absorbance was made at λ = 450 nm in semi-micro cuvettes against the blank sample.

Antioxidant activity was calculated as μM Trolox according to the following formula:

(14) $$\mathrm{AOA}=\frac{A_{450}-0.0161}{0.0018}\times K_{p},{\;\mu \mathrm{mol}\;\mathrm{Trolox}\;/\;\mathrm{dm}}^{3}$$

where: K_p_—dilution coefficient of the extract (13).

##### E. Antioxidant Activity by the ORAC (Oxygen Radical Absorbance Capacity) Method

The method developed by Ou et al. (2001) [39] with some modifications described in detail by Denev et al. (2010) [40], was applied. This method measured the ability of an antioxidant to scavange peroxyl radicals. The method is based on the inhibition in the fluorescence decline of fluorescein during its oxidation in the presence of an antioxidant. Thermal decomposition of AAPH was used as a generator of peroxyl radicals. 170 µL of fluorescein (70 nmol/L) and 10 µL of the sample were incubated for 20 min at 37 °C directly in the apparatus. Then, 20 μL of AAPH (51.5 mM final concentration) was added to the reaction mixture. The final reaction volume was 200 µL, and all solutions were prepared in phosphate buffer (75 mM, pH = 7.4). The mixture was shaken and fluorescence was read every minute until reaching zero value. To express the antioxidant activity, a standard curve with Trolox solutions (6.25 µM, 12.5 µM, 25 µM, 50 µM and 100 µM) was used. The antioxidant concentration in the sample was directly proportional to the area under the decaying fluorescence curve. The area under the attenuation fluorescence curve of a 1μM Trolox solution was assumed as one ORAC unit. The results were expressed in µmol equivalents of Trolox. The measurements were performed on a FLUOstar OPTIMA fluorimeter (BMG LABTECH, Offenburg, Germany). An excitation wavelength of 485 nm and a emission wavelenght of 520 nm were used. 

### 2.4. Methods for Mathematical and Statistical Processing

Data from triplicate experiments were processed using MS Office Excel 2013 software, using statistical functions to determine the standard deviation and maximum estimation error at significance levels of α < 0.05. The statistical analysis was performed using Statsoft Statistica 10 according to an algorithm set in the software itself. 

The grouping of malts was accomplished by the principal component analysis with the help of Statsoft Statistica 10 (Stat Soft Inc., Tulsa, OK, USA) according to an algorithm set in the software itself. 

## 3. Results

### 3.1. Main Brewing Characteristics of the Studied Malts

The results for the main brewing characteristics of the studied malt types are summarized in Table 1. According to the data provided on the manufacturer’s website, the malts were divided into three main groups—basic, special and functional malts. The basic malt types are present in the general mixture with the highest percentage, while the special and functional malt types give the beverage specific taste, aroma, color and other functional characteristics. This division is conditional, and at a later stage in the present publication we will show that there is a correlation between the malt color, respectively, the obtained wort and the observed antioxidant activity. 

All basic malt types (Table 1) had good brewing characteristics, and the yield of extract for basic malt types was in the range of 67% to 77%. The lower extract content compared to the one declared by the manufacturer [41] was due to the higher humidity due to difficulties in the storage of the samples. The increasing degree of heat treatment of the various special malts (determined by the increasing malt color) provoked a decrease in the main brewing characteristics. Since the purpose of these malt types is to give the beverage certain taste and aroma profile, they participate at a significantly lower percentage in the mixture. The main purpose of functional malts is to improve some wort qualities—pH, aroma, color, diastatic power. Their brewing characteristics were close to those of the basic malt types, but functional malt types participate in the malt mixture in relatively low quantities (up to about 10%). As the malt color is the only stable characteristic that does not change during storage, the results are presented according to the analytical evidence of each of the malt types [41].

An important characteristic of basic malts is their starch content (Table 1). For the basic malt types it varied in the range of 60.59% to 73.67%, decreasing with increasing the degree of processing of the raw material. The two main malts—Pilsner malt and wheat malt—had the highest starch content, as they had the most conservative regimes of malting and kilning and the loss of dry weight in the process was the smallest. The starch content of special malts varied widely and decreased with increasing the amount of processing of the grain. As these malts are not expected to provide the main amount of extract, their starch content is not crucial. The starch content of functional malts was relatively high and allowed for these malts to participate in larger quantities in the mixture.

### 3.2. Phenolic Compound Content and Antioxidant Activity of the Malts

**Figure 1:**
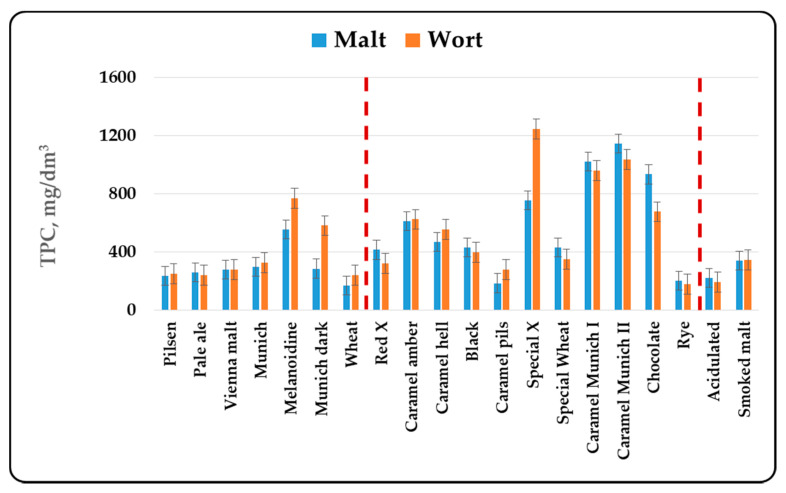
Content of total phenolic compounds in different malt types determined with the Folin-Ciocalteu reagent.

**Figure 2:**
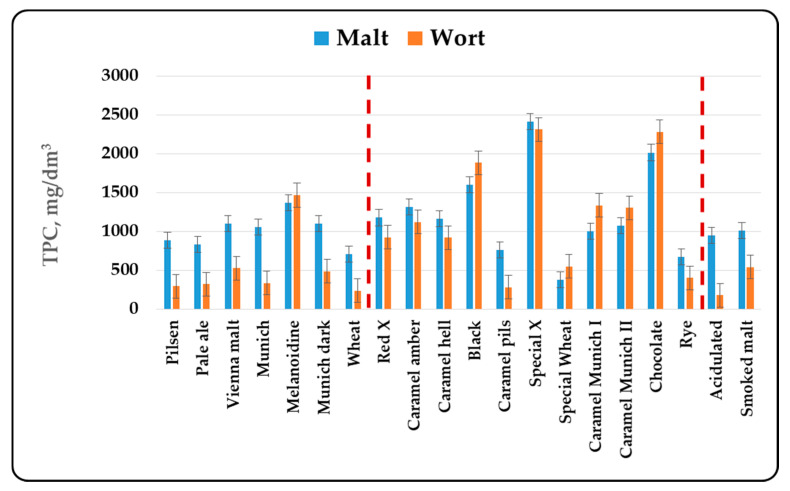
Total phenolic compounds in the different malt types, determined by the direct spectral method.

**Figure 3:**
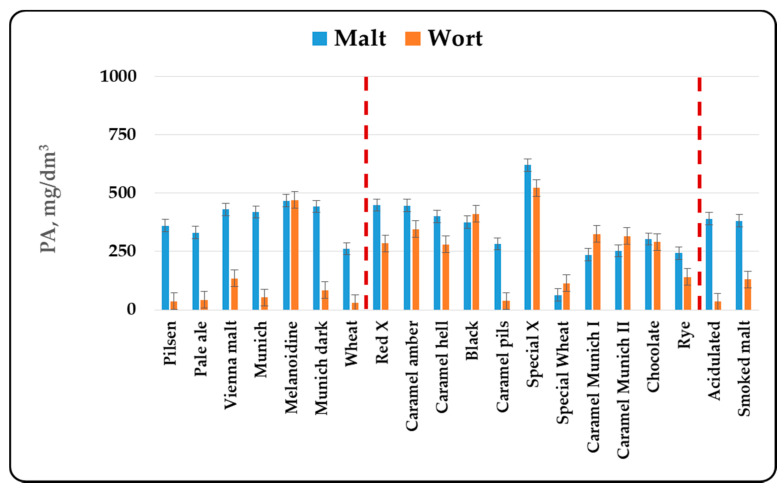
Phenolic acids in different malt types, determined by the direct spectral method.

**Figure 4:**
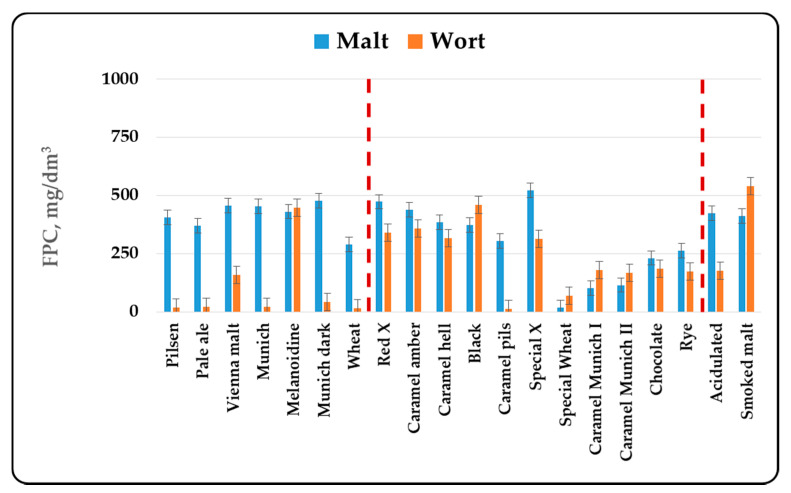
Flavonoid phenolic compounds in different malt types, determined by the direct spectral method.

In the present article, it was chosen to compare malts by the total content of phenolic components, without taking into account the individual components in malts. The reasons for this are complex. First of all, different malt types are produced from different varieties of barley. In addition, for the production of the three malt groups—basic, special and functional, different technological regimes are used, which quite naturally leads to changes in the individual components. Last but not least, the wort component composition with respect to the phenolic compounds depends on the chosen method and mode of mashing, the type and method of hopping and the mode of brewing of the wort. In this sense, the determination of the individual components of the phenolic compounds can be made by comparing production regimes or different batches of the same malt. This publication aims to compare different malt types, which requires the use of more global indicators, summarized in Figure 1, Figure 2, Figure 3 and Figure 4.

The content of phenolic compounds in malt (malt methanol extract) and wort determined by the Folin-Ciocalteu reagent are shown in Figure 1.

The content of total phenolic compounds in the basic malt types varied in the range from 1 mg/g to 1.5 mg/g (169.69–555.71 mg/dm^3^). The amount of phenolic compounds in malt increased with increasing the malt color intensity. This was due to the fact that some of the phenolic compounds polymerize and are preserved to a greater extent at moderate kilning regimes. They were usually included in the matrix of the products of the Maillard reaction and were released in the process of extraction and/or mashing, due to which a higher value of the content of phenolic compounds in darker colored malts was reported [3]. It was characteristic of the basic malt types that the entire amount of phenolic compounds passed in the wort. In the case of more highly processed malts, it was noticed that larger amount of phenolic compounds passed into the wort during mashing, which could be explained by their transition from bound (in the melanoidin matrix) to free form or the higher enzyme activity, responsible for the release of the phenolic compounds. A similar dependence was observed in wheat malt. This increase was threefold compared to the methanol extracts. Functional malts had characteristics similar to those of the basic malt types, but due to the limited number of assortments we have examined, more general conclusions were difficult to draw.

There were some differences in the group of special malts (Figure 1). There were two subgroups in it. In malts with a degree of heat treatment up to 450–500 EBC units, the tendency of increasing the phenolic content with increasing the heat treatment was retained. In this first distinct subgroup, the tendency for the release of phenolic compounds from the malt matrix during mashing was retained. In the case of special malts with a high degree of heat treatment (with a color over 500 EBC units) there was a decrease in the content of phenolic compounds, as well as a decrease in their extractability in the wort. In these malt types the enzyme systems were highly inactivated, which prevented them from acting actively and releasing phenolic compounds during mashing.

The content of total phenolic compounds, phenolic acids and flavonoid phenolic compounds was also determined by the direct spectral method. The results are presented in Figure 2, Figure 3 and Figure 4.

When determining the phenolic compounds by the direct spectral method, some of the trends were retained, but some differences were also observed. No relationship between the color and the total phenolic compounds content was observed in the basic malt types. The data show that about half of the phenolic compounds were phenolic acids and flavanoids. It has been found that only about 1/3 of these compounds were in free form and passed into the composition of the wort. It is interesting that this trend was reversed in melanoidin malt and was retained in special malt types. The data in Figure 2 show that the amount of phenolic compounds in the methanol extracts and the wort was similar, and in some cases more phenolic compounds were found in the wort. About 70–90% of the phenolic acids and the flavonoid phenolic compounds passed into the wort. The only exception was the Caramel pils malt, but if we look at its production technology, it can be said that it largely resembles that for the production of ordinary Pilsner malt. This indicated that in the special malt types the phenolic compounds were either in free form or were associated with easily extractable melanoidins and therefore a better passage into the wort was found. The change in the phenolic compounds in malt, the methanol extract and the wort in the functional malt types was similar to that in the basic malt types, because the technological production modes of both malt types are similar.

**Figure 5:**
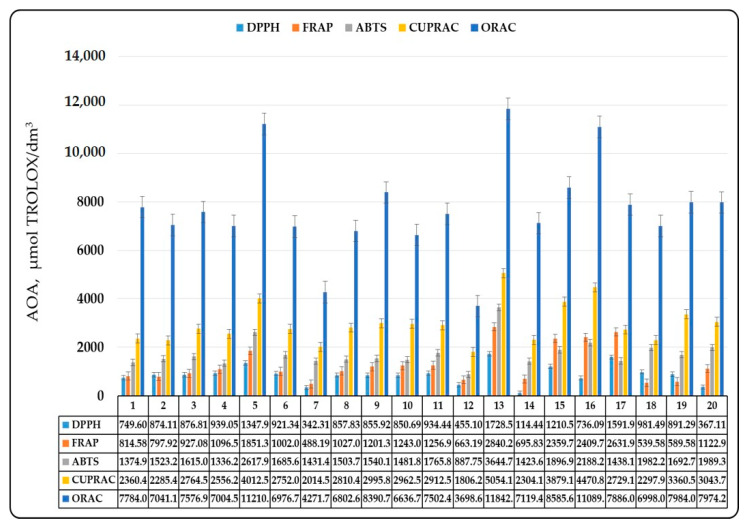
Antioxidant activity of malt. 1—Pilsen; 2—Pale ale; 3—Vienna malt; 4—Munich; 5—Melanoidine; 6—Munich dark; 7—Wheat; 8—Red X; 9—Caramel amber; 10—Caramel hell; 11—Black; 12—Caramel pils; 13—Special X; 14—Special Wheat; 15—Caramel Munich I; 16—Caramel Munich II; 17—Chocolate; 18—Rye; 19—Acidulated; 20—Smoked malt.

**Figure 6:**
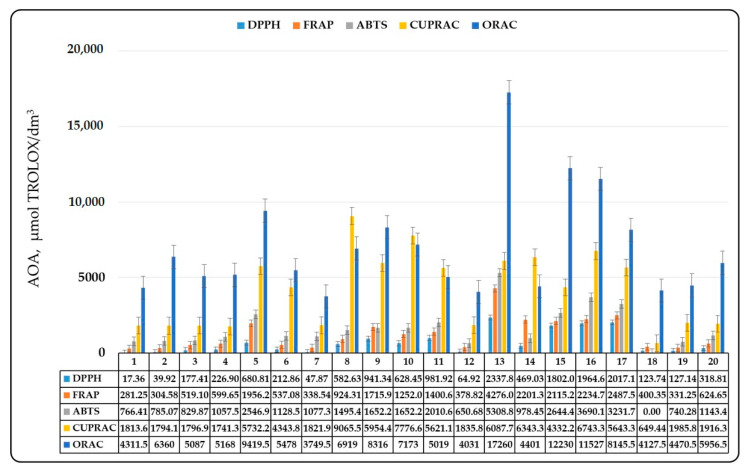
Wort antioxidant activity. 1—Pilsen; 2—Pale ale; 3—Vienna malt; 4—Munich; 5—Melanoidine; 6—Munich dark; 7—Wheat; 8—Red X; 9—Caramel amber; 10—Caramel hell; 11—Black; 12—Caramel pils; 13—Special X; 14—Special Wheat; 15—Caramel Munich I; 16—Caramel Munich II; 17—Chocolate; 18—Rye; 19—Acidulated; 20—Smoked malt.

In recent years, an important condition for the development of new types of beers and beverages based on wort is the antioxidant capacity. As it became clear from the introduction, it is determined by the content of phenolic compounds and products of the Maillard reaction in malt. The antioxidant potential of malt should be assessed by at least two different methods to obtain reliable results. In the present study, the antioxidant potential of malt was assessed by 5 different methods. The results of the study are summarized in Figure 5 for methanol malt extracts and Figure 6 for methanol wort extracts. 

The lowest antioxidant activity was registered by the DPPH method and the highest —by the ORAC method (Figure 5 and Figure 6). The results obtained indicated the existence of a clear relationship between the malt type, the content of phenolic compounds and its antioxidant activity (AOA). Basic malt types showed low AOA due to the low content of phenolic compounds and their difficulty in passing into the wort during mashing. In this case, the extraction rate did not exceed 80%, with the exception of melanoidin malt and dark Munich malt, in which the extraction rate was above 1. This could easily be explained by the fact that these two malt types had higher degree of heat treatment and the presence of more melanoidins. Therefore, AOA was due to the presence of more melanoidins in wort. The antioxidant capacity increased with the increase in the degree of heat treatment and in the malt color (Figure 5 and Figure 6). This immediately leads to the conclusion that the AOA of malt and the corresponding wort was due not only to the phenolic compounds, but also to the products of the Maillard reaction. Basic malt types showed weak inhibitory activity against the DPPH radical and higher metal-reducing ability determined by the FRAP analysis. The higher values observed by the ABTS, the CUPRAC and the ORAC methods could be explained by the different mechanisms of action of antioxidants in the presence of free radicals. However, we can assume that the basic malt types had lower biological potential because they contained fewer Maillard reaction products and phenolic compounds.

The established difficulty in the extraction of phenolic compounds in wort also led to lower biological potential of worts obtained from the basic malt types, but this should not be considered a problem, as the phenolic content in malt is responsible for the colloidal stability of beer and, therefore, for these malt types, it is better (because of other considerations) to keep the phenolic content low. In contrast to the basic malt types, there was increased antioxidant capacity in the special malt types. It was due to the increased extractability of melanoidins, other products of the Maillard reaction and phenolic compounds in wort. If a comparison of the results was made, a correlation between the content of phenolic compounds, their increased extractability in the wort and the wort AOA, can be clearly seen. In general, the degree of heat treatment also affected the AOA of the malt and its corresponding wort. The data show that highly roasted malts lost their AOA, while caramel malts with a high degree of roasting had the highest AOA (it varied between 2500 and 12,500 μmol TROLOX/dm^3^). The substances that gave malt its antioxidant potential are of particular interest. The data show that antioxidants that determined the observed activity against the DPPH radical were extracted from the malt to the least extent. In most cases, the extraction rate did not exceed 100%, although there were malts with increased extraction—mostly caramel malt type. This gave us reason to say that these substances were probably localized in the husk of the grain and during mashing remained associated with its components and were not extracted in the wort.

The results from the FRAP and the ABTS analyses were similar, again the percentage of extractability did not exceed 150%. The fact that all special malts had relatively high values of AOA compared to the CUPRAC and the ORAC method result values, and this activity was transferred to the wort, is the most impressive fact. We can assume that AOA was mainly due to the phenolic compounds and products of the Maillard reaction, which passed into the wort during mashing. It should be noted that some malts had an AOA above 7500 μmol TROLOX/dm^3^, which shows that embedded in small amounts in the mixture they would give the beer high antioxidant potential. The observed differences can be explained by the malt production processes.

The results for the functional malt types were similar to that of the basic malt types, which once again confirmed the relationship between the production method and the biological value of malt.

### 3.3. Statistical Analysis

Table 2 presents the ANOVA analysis of the results obtained for the content of phenolic compounds and the antioxidant potential of the different malt types and the wort obtained from them. ANOVA is based on the color of the malt, being the only stable feature.

The data in Table 2 (the indicators in red on a gray background) show that the degree of heat treatment (determined by the color of the malt) had a significant effect on the content of phenolic compounds of malt (determined by the FC method), the content of phenolic compounds of malt and wort (determined by the Glorie method), as well as on the content of phenolic acids and flavonoid phenolic compounds extracted in the wort. Naturally, this effect also affected most of the studied indicators of the malt antioxidant potential.

The ANOVA analysis could not be fully used to determine the effect of the treatment degree on the phenolic content and antioxidant potential of malts. For this reason, a correlation analysis of Spearman rank order correlations was performed. The correlation analysis was performed for the indicators of methanol malt extracts (Table 3) and methanol wort extracts.

The data in Table 3 show that there was a significant correlation between the malt color and the content of total phenolic compounds (determined by both methods), as well as concerning the antioxidant potential of the extracts determined by all five methods. The data also show that there was a strong correlation between the content of phenolic compounds (determined by both methods) and a large part of the antioxidant activity.

The data in Table 4 show that all the components that are responsible for its antioxidant capacity passed in the wort in the process of mashing. A strong correlation between the color of the malt and the content of phenolic compounds, on the one hand, and the wort antioxidant capacity determined by different methods, on the other hand, was found. There was also a strong correlation between the individual components of the phenolic profile and the antioxidant potential.

### 3.4. Distribution of the Malts in Different Groups as a Beginning of the Optimization of the Wort Composition

**Figure 7:**
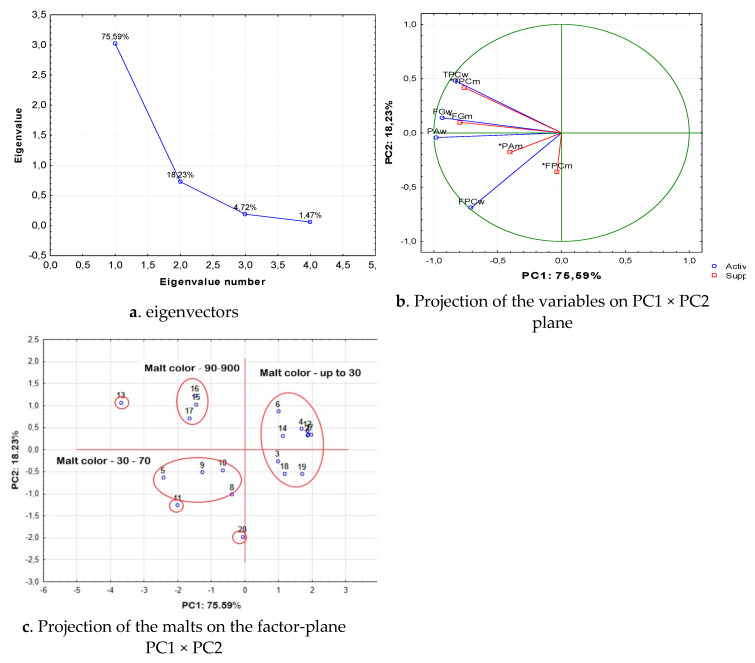
Classification of the malts by the principal components analysis with regard to the content of phenolic compounds. (**a**) eigenvectors; (**b**) Projection of the variables on PC1 × PC2 plane; (**c**) Projection of the malts on the factor-plane PC1 × PC2. 1—Pilsen; 2—Pale ale; 3—Vienna malt; 4—Munich; 5—Melanoidine; 6—Munich dark; 7—Wheat; 8—Red X; 9—Caramel amber; 10—Caramel hell; 11—Black; 12—Caramel pils; 13—Special X; 14—Special Wheat; 15—Caramel Munich I; 16—Caramel Munich II; 17—Chocolate; 18—Rye; 19—Acidulated; 20—Smoked malt. Legend: “,”—decimal point; “-”—minus sign; *—denotes the supplement variable during classification.

**Figure 8:**
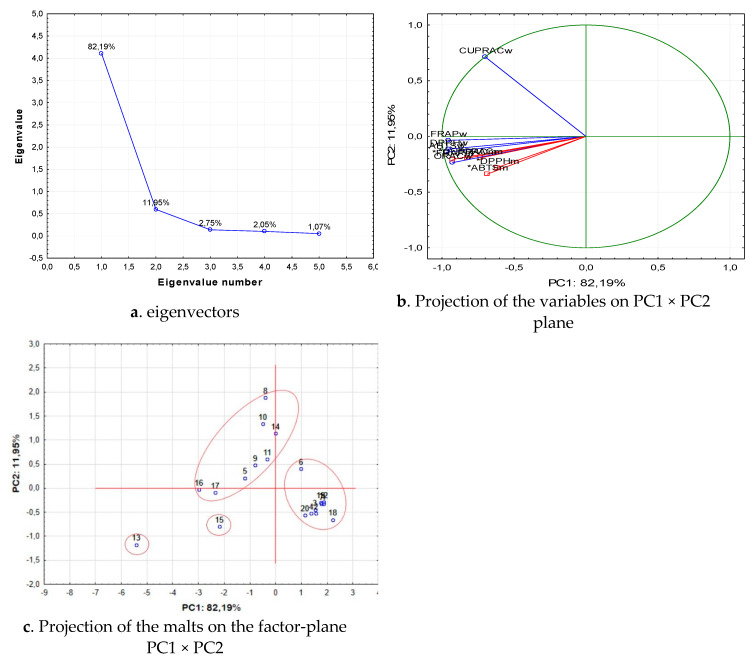
Classification of malts by the principal component analysis in terms of antioxidant potential. (**a**) eigenvectors; (**b**) Projection of the variables on PC1 × PC2 plane; (**c**) Projection of the malts on the factor-plane PC1 × PC2. 1—Pilsen; 2—Pale ale; 3—Vienna malt; 4—Munich; 5—Melanoidine; 6—Munich dark; 7—Wheat; 8—Red X; 9—Caramel amber; 10—Caramel hell; 11—Black; 12—Caramel pils; 13—Special X; 14—Special Wheat; 15—Caramel Munich I; 16—Caramel Munich II; 17—Chocolate; 18—Rye; 19—Acidulated; 20—Smoked malt. Legend: “,”—decimal point; “-”—minus sign; *—denotes the supplement variable during classification.

The distribution of the malts into groups by the principal component analysis was carried out by the data for the phenolic compounds extracted in the wort. The data for the phenolic compounds of malts was included as additional data. The results are presented in Figure 7. The second group of parameter values, used for the classification of malts, was their antioxidant capacity (Figure 8).

The data set was divided by two eigenvectors, which made up to 93.8% of the total amount of data (Figure 7a). The data show that malts were mainly distributed in the groups with respect to two components—phenolic acids and flavonoid phenolic compounds (Figure 7b). Total phenolic compounds had less effect on the distribution of the malts in the individual groups. The distribution of the malts with respect to the two main components is shown in Figure 7c. As can be seen, malts were distributed into three main groups relative to the two main components. The first group was located to the right of the first principal component in the first and second quadrant, but was relatively homogeneous. If we compare the malts that were included in it and the malt color, shown in Table 1, it could be seen that it contained all malts with a color intensity of up to 30 units. This group included 10 malts. An exception was the Smoked malt, which also had low coloration, but did not fall into this group. The second malt group was located in the fourth quadrant, and it included 3 malts with color intensity between 90 and 900 units. The third malt group was located in the third quadrant of the factor space and was characterized by color intensity in the range of 30 to 70 units. The single malts, which had higher color intensity, were located outside of the three groups. In general, this distribution of malts confirmed the data from the literature (see Discussion) regarding the relationship between the malt color and the malt content of phenolic compounds.

The distribution of malts in groups in terms of their antioxidant capacity shows that once again there were two main components, which represented over 93% of the variants (Figure 8a). The values of the wort AOA, determined by the FRAP and the ABTS methods, were of fundamental importance for the malt distribution. The values of the wort AOA, determined by the FRAP and the ABTS methods, had the highest absolute values of the eigenvectors on PC1 (Figure 8b). The results for the classification of malts with respect to the AOA of the wort obtained from them show that the malts were divided into two main large groups (Figure 8c). The first group included basic malts (1, 2, 3, 4, 6, 7), special malts (12—Caramel pils), the rye malt and both functional malts. The second large group included one of the basic malts (6—Melanoidin) and all special malts without 13—Special X. Even though slightly distant from the other malts, 15—Caramel Munich I could belong to the group of special malts, not only from brewery point of view, but also from a statistical point of view. The applied statistical processing proved that the malts were actually distributed into two main groups with regard to the AOA. The basic malt types had lower AOA, which was determined by the lower content of products of the Maillard reaction. This was evidenced by the fact that melanoidin malt, which was a basic malt with higher degree of heat treatment from a statistical point of view, found a place in the group of special malts; meanwhile the less colored 12—Caramel pils malt was part of the basic malt types group. The second group—special malts—had higher AOA, which was determined by the products of the Maillard reaction. 

## 4. Discussion

The results obtained from the study of different malt types were in agreement with the data cited in the specialized literature. In the first place, changes in the content of phenolic compounds were due to the malting process [3,42]. The increase in malt antioxidant activity was due to the enzymatic release of phenolic compounds during malting [3,12,13]. Essential for the formation of the biological activity of malts was their heat treatment— kilning and roasting. The data from our study (Figure 1, Figure 2, Figure 3 and Figure 4) show that as the color increased to about 400–500 units, the content of phenolic compounds in malt increased as well. Antioxidant activity has been shown to increase during kilning due to increased amounts of polyphenols in malt [3,43]. This was due to the higher levels of ferulic acid in the heat-treated malts. They increased between 2 and 7 times [17] and were responsible for the higher antioxidant capacity of dark beer types [44]. In highly roasted malt types (with a color over 500 units) there was a tendency to reduce the content of phenolic compounds, which confirmed the observations of other authors [45]. This was related to the low levels of catechin and ferulic acid, which were responsible for the reduction of the antioxidant capacity during steeping and germination [11]. Low AOA has also been found to be due to low levels of catechin, prodelphinin B3 and procyanidin B3 [8]. The method of grinding, as well as the chosen method and mode of mashing were also important for the content of phenolic compounds in wort and beer [3,46,47,48,49].

The data obtained also confirmed the relationship between the AOA of the malt and the wort obtained from it and the color of the beer [3,50]. The higher antioxidant activity of malts was mainly due to the products formed in the Maillard reaction, which correlated with the malt color and the content of melanoidins [3,43,51]. Catechins, caffeic acid, ferulic acid and sinapic acid are major contributors to AOA [3,30]. Catechin, for example, has higher metal-reducing ability, while ferulic acid has higher DPPH radical inhibitory ability [3,11]. In a study of the DPPH reducing ability, it was found that barley phenols had low AOA, but after undergoing heat treatment AOA increased significantly [3,51,52]. The obtained experimental data were also confirmed by the studies of Sharma and Gujral (2011) [53] and Cechovska et al. (2012) [7], which show that the presence of products of the Maillard reaction increased malt AOA up to 13 times.

In addition to the numerous phenolic compounds, aromatic amino acids also have antioxidant properties. According to Spreng and Hofmann (2018) [54], the content of phenylalanine, tyrosine and tryptophan correlates significantly with the ORAC values; tyrosine content with the FRAP values in the different barley malts.

The development of new types of beer and wort-based beverages should be subject to knowledge of both the malt brewing characteristics and the knowledge of the biological capacity of the malt that is included in the preparation of the beverage. The development of new types of beverages should be based on the development of mixtures, using the methods of mathematical modeling of mixtures and the composition of mixtures should be determined on the following principles: at least 50% of the composition of the meal should be occupied by one of the basic malt types; the remaining 50% should be distributed between special and functional malt types. The reasons for this distribution are due to the fact that special malt types, especially those with higher degree of heat treatment, cannot provide the necessary enzyme systems responsible for hydrolysis during wort production. Even though special malts provide higher antioxidant potential, the lack of enzyme systems is leading in terms of the formulation of the wort composition.

## 5. Conclusions

Our results showed that there was a relationship between malt color and the content of phenolic compounds, as well as between the content of the Maillard reaction products and malt biological activity. The malts with the highest degree of heat treatment were characterized by the highest antioxidant activity, which was due to the Maillard reaction products with antioxidant capacity formed in them. These results, especially in the grouping of malts in different groups in terms of phenolic content and antioxidant potential, were the basis for the process of modeling the composition of the malt meal. They allow, on the basis of knowledge of the malt biological value and its brewing characteristics, including the organoleptic characteristics of the malt and the resulting wort, to develop mixtures intended for the preparation of a specific type of beverage, i.e., to apply the so-called tailor-made concept for the production of the beverage.

The established correlation between the malt color and its biological value could be applied to assess the malt biological potential and is a quick method for selection of malts in the process of modeling the composition of the beverage. 

The obtained results confirm the observations of other authors. The purpose of the publication is for the results to serve as a scientific basis for modeling the wort composition, using the phenolic content and the antioxidant capacity of malt as target functions. Naturally, these results will be the subject of other future publications.

## Figures and Tables

**Table 1:** Main brewing characteristics of the studied malts.

No.	Malt Type	Wort Extract, °P	Extract Yield, %		Malt Color, EBC Units * [40]	Moisture, %	Starch, %
			AirDW	AbsDW			
Basic Malt Types							
1	Pilsen	8.22 ± 0.32	71.87 ± 0.58	75.66 ± 0.58	3–4.9	5.37 ± 0.14	66.64 ± 0.06
2	Pale ale	8.32 ± 0.25	72.80 ± 0.39	76.63 ± 0.39	5–7	5.12 ± 0.19	61.66 ± 0.59
3	Vienna	8.31 ± 0.12	72.68 ± 0.33	76.51 ± 0.33	8–10	5.78 ±0.23	65.47 ± 0.26
4	Munich	8.07 ± 0.21	70.45 ± 0.29	74.15 ± 0.29	11–20	5.30 ± 0.58	65.21 ± 0.17
5	Melanoidin	8.01 ± 0.16	69.90 ± 0.59	73.58 ± 0.59	61–80	4.75 ± 0.22	61.89 ± 0.66
6	Munich dark	8.63 ± 0.33	75.84 ± 0.63	79.83 ± 0.63	21–35	5.33 ± 0.13	60.59 ± 0.87
7	Wheat	7.39 ± 0.18	64.04 ± 0.68	67.41 ± 0.68	3.5–6	4.89 ± 0.34	73.67 ± 0.13
Special Malt Types							
8	Red X	7.83 ± 0.22	68.15 ± 0.63	71.74 ± 0.63	28–32	5.57 ± 0.08	63.38 ± 0.28
9	Caramel amber	5.67 ± 0.14	48.21 ± 0.45	50.75 ± 0.45	61–80	5.78 ± 0.37	58.14 ± 0.55
10	Caramel hell	6.91 ± 0.37	59.52 ± 0.33	62.66 ± 0.33	20–40	4.37 ± 0.13	59.58 ± 0.99
11	Black	3.12 ± 0.08	25.83 ± 0.22	27.19 ± 0.22	1100–1200	4.13 ± 0.04	30.18 ± 2.42
12	Caramel pils	6.34 ± 0.18	54.32 ± 0.56	57.18 ± 0.56	3–7	5.53 ± 0.53	63.05 ± 0.83
13	Special X	4.09 ± 0.09	34.22 ± 0.48	36.02 ± 0.48	300–400	4.33 ± 0.26	17.19 ± 1.07
14	Special Wheat	7.54 ± 0.18	65.44 ± 0.58	68.89 ± 0.58	16–20	4.21 ± 0.15	66.68 ± 0.08
15	Caramel Munich I	6.73 ± 0.21	57.91 ± 0.59	60.95 ± 0.59	81–100	3.96 ± 0.10	53.24 ± 1.50
16	Caramel Munich II	5.67 ± 0.14	48.24 ± 0.65	50.78 ± 0.65	110–130	4.48 ± 0.43	15 ± 1.48
17	Chocolate	4.15 ± 0.07	34.75 ± 0.45	36.57 ± 0.45	800–1000	2.80 ± 0.11	7.08 ± 0.85
18	Rye malt	7.87 ± 0.21	68.50 ± 0.39	72.11 ± 0.39	-	5.90 ± 0.14	60.89 ± 0.82
Functional Malt Types							
19	Acidulated	2.33 ± 0.09	18.8 ± 0.23	19.8±0.23	3–8	4.95 ± 0.67	61.85 ± 0.38
20	Smoked	8.42 ± 0.21	73.2 ± 0.45	77.0±0.45	3–8	5.99 ± 0.35	59.93 ± 0.71

* the color of malt or wort are presented according to the manufacturer data; AirDW—air dry weight; AbsDW—absolutely dry weight; °P—degree Plato; “-”—not measured.

**Table 2:** Analysis of variances (ANOVA).

Analysis of Variance Marked Effects are Significant at *p* < 0.05000								
	SS Effect	df Effect	MS Effect	SS Error	df Error	MS Error	F	*p*
TPCm	1078156	10	107816	2726	2	1363	79.11	0.012545
TPCw	1251217	10	125122	27260	2	13630	9.18	0.102161
FGm	3699327	10	369933	8485	2	4242	87.20	0.011389
FGw	6763666	10	676367	96	2	48	14026.40	0.000071
PAm	207003	10	20700	4218	2	2109	9.81
PAw	289436	10	28944	9	2	5	6261.14	0.000160
FPCm	254477	10	25448	8758	2	4379	5.81	0.155646
FPCw	249845	10	24985	347	2	173	144.15	0.006908
DPPHm	2000181	10	200018	43389	2	21694	9.22	0.101746
DPPHw	8293012	10	829301	2181	2	1090	760.49	0.001314
FRAPm	7486926	10	748693	34781	2	17391	43.05	0.022908
FRAPw	16919969	10	1691997	58479	2	29240	57.87	0.017103
ABTSm	5007264	10	500726	118931	2	59466	8.42	0.110744
ABTSw	26742097	10	2674210	18987	2	9494	281.69	0.003543
CUPRACm	9604245	10	960424	165115	2	82558	11.63	0.081698
CUPRACw	87979953	10	8797995	830864	2	415432	21.18	0.045910
ORACm	40018408	10	4001841	8359008	2	4179504	0.96	0.612666
ORACw	168293859	10	16829386	71598	2	35799	470.11	0.002124

**Table 3:** Correlation analysis of methanol malt extracts.

Spearman Rank Order Correlations Marked Correlations are Significant at *p* < 0.05000										
	Color	TPCm	FGm	PAm	FPCm	DPPHm	FRAPm	ABTSm	CUPRACm	ORACm
**Color**	1.000000	0.879097	0.754519	0.199548	−0.003012	0.568525	0.853164	0.454820	0.597892	0.483434
**TPCm**	-	1.000000	-	-	-	0.337721	0.916134	0.422715	0.713802	0.641595
**FGm**	0.754519	-	1.000000	-	-	0.551880	0.807519	0.374436	0.622556	0.427068
**PAm**	0.199548	-	-	1.000000		0.345865	0.312782	0.233083	0.433083	0.192481
**FPCm**	−0.003012	-	-	-	1.000000	0.275188	0.109774	0.157895	0.287218	0.066165

“-”—not measured.

**Table 4:** Correlation analysis of methanol wort extracts.

Spearman Rank Order Correlations Marked Correlations Are Significant at *p* < 0.05000										
	Color	TPCw	FGw	PAw	FPCw	DPPHw	FRAPw	ABTSw	CUPRACw	ORACw
**Color**	1.000000	0.816266	0.932230	0.893826	0.612199	0.925453	0.860694	0.839925	0.631778	0.740212
**TPCw**	-	1.000000	-	-	-	0.893233	0.890226	0.913125	0.669173	0.822556
**FGw**	-	-	1.000000	-	-	0.933835	0.908271	0.883039	0.648120	0.769925
**PAw**	-	-	-	1.000000	-	0.873684	0821053	0.796540	0.574436	0.760902
**FPCw**	-	-	-	-	1.000000	0.675188	0.559398	0.591200	0.529323	0.560902

“-”—not measured.

## Data Availability

Data is contained within the article.

## References

[1] Eaton B., Priest D., Stewart G. (2006). An overview of brewing. Handbook of Brewing.

[2] Kunze W. (2004). Technology of Brewing and Malting.

[3] Carvalho D.O., Correia E., Lopes L., Guido L.F. (2014). Further insights into the role of melanoidins on the antioxidant potential of barley malt. Food Chem..

[4] Vanderhaegen B., Neven H., Verachtert H., Derdelinckx G. (2006). The chemistry of beer aging—A critical review. Food Chem..

[5] De Keukeleire D. (2000). Fundamentals of beer and hop chemistry. Quim. Nova.

[6] Quifer-Rada P., Vallverdú-Queralt A., Martínez-Huélamo M., Chiva-Blanch G., Jáuregui O., Estruch R., Lamuela-Raventós R. (2015). A comprehensive characterisation of beer polyphenols by high resolution mass spectrometry (LC–ESI-LTQ-Orbitrap-MS). Food Chem..

[7] Čechovská L., Konečný M., Velíšek J., Cejpek K. (2012). Effect of Maillard reaction on reducing power of malts and beers. Czech J. Food Sci..

[8] Leitao C., Marchioni E., Bergaentzle M., Zhao M., Didierjean L., Miesch L., Holder E., Miesch M., Ennahar S. (2012). Fate of polyphenols and antioxidant activity of barley throughout malting and brewing. J. Cereal Sci..

[9] Holtekjølen A.K., Kinitz C., Knutsen S.H. (2006). Flavanol and Bound Phenolic Acid Contents in Different Barley Varieties. J. Agric. Food Chem..

[10] Madhujith T., Izydorczyk A.M., Shahidi F. (2006). Antioxidant Properties of Pearled Barley Fractions. J. Agric. Food Chem..

[11] Lu J., Zhao H., Chen J., Fan W., Dong J., Kong W., Sun J., Cao Y., Cai G. (2007). Evolution of Phenolic Compounds and Antioxidant Activity during Malting. J. Agric. Food Chem..

[12] Dvořáková M., Guido L.F., Dostalek P., Skulilová Z., Moreira M.M., Barros A.A. (2008). Antioxidant Properties of Free, Soluble Ester and Insoluble-Bound Phenolic Compounds in Different Barley Varieties and Corresponding Malts. J. Inst. Brew..

[13] Dvorakova M., Moreira M.M., Dostalek P., Skulilova Z., Guido L.F., Barros A.A. (2008). Characterization of monomeric and oligomeric flavan-3-ols from barley and malt by liquid chromatography–ultraviolet detection–electrospray ionization mass spectrometry. J. Chromatogr. A.

[14] Magalhães P.J., Almeida S.M., Carvalho A.M., Gonçalves L.M., Pacheco J.G., Cruz J.M., Guido L.F., Barros A.A. (2011). Influence of malt on the xanthohumol and isoxanthohumol behavior in pale and dark beers: A micro-scale approach. Food Res. Int..

[15] Goupy P., Hugues M., Boivin P., Amiot M.J. (1999). Antioxidant composition and activity of barley (*Hordeum vulgare*) and malt extracts and of isolated phenolic compounds. J. Agric. Food Chem..

[16] Samaras T.S., Camburn P.A., Chandra S.X., Gordon M.H., Ames J.M. (2005). Antioxidant Properties of Kilned and Roasted Malts. J. Agric. Food Chem..

[17] Inns E.L., Buggey L.A., Booer C., Nursten H.E., Ames J.M. (2007). Effect of Heat Treatment on the Antioxidant Activity, Color, and Free Phenolic Acid Profile of Malt. J. Agric. Food Chem..

[18] Inns E.L., Buggey L.A., Booer C., Nursten H.E., Ames J.M. (2011). Effect of Modification of the Kilning Regimen on Levels of Free Ferulic Acid and Antioxidant Activity in Malt. J. Agric. Food Chem..

[19] Maillard M.-N., Berset C. (1995). Evolution of Antioxidant Activity during Kilning: Role of Insoluble Bound Phenolic Acids of Barley and Malt. J. Agric. Food Chem..

[20] Coghe S., Adriaenssens B., Leonard S., Delvaux F.R. (2004). Fractionation of Colored Maillard Reaction Products from Dark Specialty Malts. J. Am. Soc. Brew. Chem..

[21] Coghe S., D’Hollander H., Verachtert H., Delvaux F.R. (2005). Impact of Dark Specialty Malts on Extract Composition and Wort Fermentation. J. Inst. Brew..

[22] Coghe S., Gheeraert B., Michiels A., Delvaux F.R. (2006). Development of Maillard Reaction Related Characteristics During Malt Roasting. J. Inst. Brew..

[23] Yahya H., Linforth R.S.T., Cook D. (2014). Flavour generation during commercial barley and malt roasting operations: A time course study. Food Chem..

[24] Morales F.J., Fernandez-Fraguas C., Jimenez-Perez S. (2005). Iron-binding ability of melanoidins from food and model systems?. Food Chem..

[25] Wang H.-Y., Qian H., Yao W.-R. (2011). Melanoidins produced by the Maillard reaction: Structure and biological activity. Food Chem..

[26] Guido L.F., Fortunato N.A., Rodrigues J.A., Barros A.A. (2003). Voltammetric Assay for the Aging of Beer. J. Agric. Food Chem..

[27] Landete J.M. (2013). Dietary Intake of Natural Antioxidants: Vitamins and Polyphenols. Crit. Rev. Food Sci. Nutr..

[28] Subba Rao M.V., Muralikrishna G. (2002). Evaluation of the Antioxidant Properties of Free and Bound Phenolic Acids from Native and Malted Finger Millet (Ragi, *Eleusine coracana* Indaf-15). J. Agric. Food Chem..

[29] Zhao H., Chen W., Lu J., Zhao M. (2010). Phenolic profiles and antioxidant activities of commercial beers. Food Chem..

[30] Leitao C., Marchioni E., Bergaentzlé M., Zhao M., Didierjean L., Taidi B., Ennahar S. (2011). Effects of Processing Steps on the Phenolic Content and Antioxidant Activity of Beer. J. Agric. Food Chem..

[31] Jolliffe I.T., Cadima J. (2016). Principal component analysis: A review and recent developments. Philos. Trans. R. Soc. A Math. Phys. Eng. Sci..

[32] Analytica (Version 2019)—European Brewing Convention. https://brewup.eu/ebcanalytica.

[33] Luckanov N., Ivanova T., Pishtiyski I., Koleva A. (1994). Biochemistry (Laboratory Manual).

[34] Mazza G., Fukumoto L., Delaquis P., Girard B., Ewert B. (1999). Anthocyanins, Phenolics, and Color of Cabernet Franc, Merlot, and Pinot Noir Wines from British Columbia. J. Agric. Food Chem..

[35] Dinkova R., Heffels P., Shikov V., Weber F., Schieber A., Mihalev K. (2014). Effect of enzyme-assisted extraction on the chilled storage stability of bilberry (*Vaccinium myrtillus* L.) anthocyanins in skin extracts and freshly pressed juices. Food Res. Int..

[36] Benzie I.F.F., Strain J.J. (1996). The ferric reducing ability of plasma (FRAP) as a measure of “antioxidant power”: The FRAP assay. Anal. Biochem..

[37] Iqbal E., Abu Salim K., Lim L.B. (2015). Phytochemical screening, total phenolics and antioxidant activities of bark and leaf extracts of Goniothalamus velutinus (Airy Shaw) from Brunei Darussalam. J. King Saud Univ. Sci..

[38] Apak R., Güçlü K., Özyürek M., Karademir S.E., Erçağ E. (2006). The cupric ion reducing antioxidant capacity and polyphenolic content of some herbal teas. Int. J. Food Sci. Nutr..

[39] Ou B., Hampsch-Woodill M., Prior R.L. (2001). Development and Validation of an Improved Oxygen Radical Absorbance Capacity Assay Using Fluorescein as the Fluorescent Probe. J. Agric. Food Chem..

[40] Denev P., Ciz M., Ambrozova G., Lojek A., Yanakieva I., Kratchanova M. (2010). Solid-phase extraction of berries’ anthocyanins and evaluation of their antioxidative properties. Food Chem..

[41] BestMalz Catalog. https://bestmalz.de/.

[42] Sharma P., Gujral H.S. (2010). Antioxidant and polyphenol oxidase activity of germinated barley and its milling fractions. Food Chem..

[43] Chandra C.S., Buggey L.A., Peters S., Cann C., Liegeois C. (2001). Factors Affecting the Development of Antioxidant Properties of Malts during the Malting and Roasting Process.

[44] Piazzon A., Forte M., Nardini M. (2010). Characterization of Phenolics Content and Antioxidant Activity of Different Beer Types. J. Agric. Food Chem..

[45] Kunz T., Muller C., Mato-Gonzales D., Methner F.-J. (2012). The influence of unmalted barley on the oxidative stability of wort and beer. J. Inst. Brew..

[46] Szwajgier D. (2011). Dry and Wet Milling of Malt. A Preliminary Study Comparing Fermentable Sugar, Total Protein, Total Phenolics and the Ferulic Acid Content in Non-Hopped Worts. J. Inst. Brew..

[47] Vanbeneden N., Gils F., Delvaux F., Delvaux F.R. (2007). Variability in the Release of Free and Bound Hydroxycinnamic Acids from Diverse Malted Barley (*Hordeum vulgare* L.) Cultivars during Wort Production. J. Agric. Food Chem..

[48] Vanbeneden N., Van Roey T., Willems F., Delvaux F., Delvaux F.R. (2008). Release of phenolic flavour precursors during wort production: Influence of process parameters and grist composition on ferulic acid release during brewing. Food Chem..

[49] Bartolome B., Garcia-Conesa M.T., Williamson G. (1996). Release of the bioactive compound, ferulic acid, from malt extracts. Biochem. Soc. Trans..

[50] Polak J., Bartoszek M., Stanimirova I. (2013). A study of the antioxidant properties of beers using electron paramagnetic resonance. Food Chem..

[51] Coghe S., Vanderhaegen B., Pelgrims B., Basteyns A.-V., Delvaux F.R. (2003). Characterization of Dark Specialty Malts: New Insights in Color Evaluation and Pro- and Antioxidative Activity. J. Am. Soc. Brew. Chem..

[52] Papetti A., Daglia M., Aceti C., Quaglia M., Gregotti C., Gazzani G. (2006). Isolation of an in Vitro and ex Vivo Antiradical Melanoidin from Roasted Barley. J. Agric. Food Chem..

[53] Sharma P., Gujral H.S. (2011). Effect of sand roasting and microwave cooking on antioxidant activity of barley. Food Res. Int..

[54] Spreng S., Hofmann T. (2018). Activity-Guided Identification of in Vitro Antioxidants in Beer. J. Agric. Food Chem..

